# Determination of the optimal dose and dosing strategy for effective l-menthol oral rinsing during exercise in hot environments

**DOI:** 10.1007/s00421-024-05609-w

**Published:** 2024-10-05

**Authors:** Owen Jeffries, Godi Jibi, Joe Clark, Martin Barwood, Mark Waldron

**Affiliations:** 1 School of Biomedical, Nutritional and Sport Sciences, Newcastle University, Newcastle Upon Tyne, NE2 4HH UK; 2 Department of Sport, Health and Nutrition, Leeds Trinity University, Leeds, UK; 3 College of Engineering, Swansea University, Swansea, UK

**Keywords:** Thermoregulation, Perception, Menthol, Heat, Dose–response, Performance

## Abstract

**Purpose:**

This multi-study programme investigated the optimal concentration of l-menthol delivered as an oral mouth rinse to modulate thermo-behaviour during exercise in a hot environment (35 °C).

**Method:**

In study 1, 38 participants completed a survey to establish an effective and tolerable range of l-menthol concentration. 31 participants completed an RPE-protocol examining 1. the dose–response effect of l-menthol mouth rinse on exercise performance (*n* = 16) and 2. the temporal effectiveness of administering l-menthol in an incremental and decremental dosing pattern (*n* = 15). Power output, heart rate, body core temperature and thermal sensation were reported throughout.

**Results:**

The optimal menthol concentration for peak power was between 0.01 and 0.1% (~ 6% increase, *P* < 0.05) and 0.5% (~ 9% increase, *P* < 0.05) with respect to control. Work completed was increased at 0.01% (~ 5%, *P* < 0.05), at 0.1% (~ 3%, *P* < 0.05) and had a detrimental effect at 0.5% (− 10% decrease, *P* < 0.05). There were no differences between an ascending dose protocol (0.01 to 0.5%), descending dose protocol (0.5–0.01%) or a constant 0.01% dose protocol. There were no reported differences in body core temperature or heart rate across trials (*P* > 0.05).

**Conclusion:**

The optimal dose of l-menthol when delivered via oral rinsing is between 0.01 and 0.1%. At lower concentrations, l-menthol appears to be less effective and at higher concentrations (> 0.5%) l-menthol appears to elicit greater irritation and may not positively modulate thermo-behaviour during exercise in a hot environment.

## Introduction

Somatosensory afferent fibres located in the oral cavity are activated by a range of stimuli that provide information on sensations in the mouth, which include nociception, mechanosensation and thermal state. The transduction of sensory information by trigeminal nerve fibres to the brain is mediated via activation of transient receptor potential (TRP) ion channels. Whilst the TRP vanilloid (TRPV1) and TRP ankyrin (TRPA1) channels are sensitive to heat/capsaicin and irritants/mustard oil, triggering sensations of warmth and/or irritation (Julius 2013), the sensation of oral cooling is transduced by the TRP melastatin 8 (TRPM8) channel. TRPM8 is modulated by cooling temperatures (range 8–28 °C) or mimetics such as l-menthol (Andersen et al. 2014; Bautista et al. 2007; McKemy et al. 2002; Peier et al. 2002). However, l-menthol elicits multimodal somatosensory sensations that include cooling but also sensations of irritation and tingling (Cliff and Green 1994a, b; Dessirier et al. 2001; Green 1992; Green and McAuliffe 2000). Whilst l-menthol’s sensations of coolness are primarily associated with TRPM8, in higher concentrations, irritation, tingling and even burning or painful sensations may be mediated by its interaction with TRPA1 or TRPV1, which are better known for their role in pain sensations (Karashima et al. 2007; Takaishi et al. 2016).

Interest in l-menthol’s perceived cooling properties to facilitate more favourable thermal perception in hot environments have seen it tested against range of exercise modalities (Barwood et al. 2020). The consensus is that oral rinsing or ingestion is the most efficacious strategy to enhance exercise performance (Jeffries and Waldron 2019). However, the concentration of l-menthol applied during oral rinsing varies across the literature from 0.01% (Flood et al. 2017; Jeffries et al. 2018; Mündel and Jones 2010; Stevens et al. 2016) to 0.1% (Crosby et al. 2022; Saldaris et al. 2020). However, the effective dose range required to elicit an optimal human thermo-behavioural response is unclear. Further, no clear guidelines currently exist on the optimal dose of l-menthol that should be applied during oral rinsing, as we previously established in a Delphi consensus report, extending across the breadth of the literature (Barwood et al. 2020). In studies that investigate taste, oral rinsing of l-menthol at concentrations < 0.03% generally report that coolness is the dominant sensation; however, at higher concentrations (> 0.3%), the predominant sensation is that of irritation (Cliff and Green 1994a, b), thus suggesting that l-menthol may have a confined operation range to elicit favourable perceptual cooling sensations.

We have previously reported an observed loss in potency across a series (> 2 swills) of oral l-menthol applications when administered at 10-min intervals. Here, following an initial performance enhancement and improved cooling perception in the heat with l-menthol administration, a progressive decrease in perceptually controlled cycling performance was observed after the second mouth rinse (Flood et al. 2017). We postulated two explanations for this: (i) l-menthol may have limited potency during periods of high internal thermal strain or that (ii) l-menthol may have lost its effectiveness as a function of the number of oral applications. We furthered this work to demonstrate that orally rinsed l-menthol remains potent during periods of high internal heat strain (> 38.5 °C) (Jeffries et al. 2018), increasing exercise capacity to a comparable amount as crushed ice. However, it remains unclear following an initial application of l-menthol, whether subsequent applications can still be effective. Molecular evidence would suggest that TRPM8 channels are desensitised for a period of up to 10 min following l-menthol application (McKemy et al. 2002); however, once a dose range is established, it is possible that modifying the dose could extend its cooling and performance enhancing effects, beyond that reported in response to a single oral rinse.

The purpose of the present multi-study programme was, therefore, to establish clear evidence for l-menthol’s thermo-behavioural effective dose during exercise in a hot environment, in humans. In experiment 1, a participant group was surveyed regarding perception of coolness and irritation to establish an effective and tolerable range of l-menthol concentration. In experiment 2, we aimed to establish a dose response curve for thermo-behavioural responses to l-menthol when exercising in a hot environment. Finally, in experiment 3, the temporal effectiveness of administering a range of l-menthol concentrations in an incremental and decremental dosing pattern when exercising in the heat was explored. Of primary interest was the ability to discriminate an effective range of l-menthol concentrations to optimise application and facilitate the development of recommended guidelines.

## Materials and methods

### Participants

Data were gathered from 38 male participants (age = 36 ± 3 years) on taste characteristics of a range of l-menthol concentrations in a randomised, double-blind, crossover survey. In subsequent experimental trials, 31 healthy non-acclimated males (age = 21 ± 2 years; body mass = 79.1 ± 9.2 kg; stature = 180.3 ± 6.7 cm) were recruited to participate in two independent randomised, double-blind, crossover experimental studies, exploring the dose–response eff1ect of l-menthol mouth rinse on exercise performance (study 2, *n* = 16) or, in a follow-up study, administration of increasing or decreasing doses of l-menthol on exercise performance (Study 3, *n* = 15). Randomisation was conducted by generating random numbers for each condition for all participants using online software (Urbaniak and Plous 2015) and blinding was performed by a person that was not on the experimental research team and all solutions were administered with random letters. Participants were blinded to the original hypothesis of the study and informed that the effect of differing mouth rinses on exercise in the heat was being investigated. Participants abstained from alcohol, caffeine, and strenuous exercise in the 24 h leading up to the day of testing. None of the participants had visited a hot country in the previous three months and all testing was conducted during the UK winter months of November to April. Participants visited the laboratory on between 5 and 6 separate occasions, each separated by at least 72-h. A-priori sample size was calculated using G*Power (version 3.1.9.6). Given the effect size (*η*_*p*_^*2*^ = 0.896; (Flood et al. 2017)), we reported previously for differences in power output using an RPE-16 protocol with l-menthol, a sample size of 10 was deemed sufficient to identify differences between groups with a statistical power of 0.95. More participants were recruited to account for experimental attrition. Informed written consent was obtained from each participant before commencing the study. Ethical approval was provided by the Newcastle University ethics committee.

### Experimental procedures

In study 1, participants were asked to orally rinse a range of l-menthol concentrations (0.064 mM = 0.001% to 64 mM = 1%) delivered at a temperature of ~ 31 °C, equivalent to oral temperature neutrality (Green 1985). Laboratory conditions were maintained at 20 ± 0.6 °C and testing was controlled at the same time of day ± 2 h. Participants rinsed 25 ml solutions for 10 s before expectorating into a bucket, they were told to keep their mouth closed to prevent evaporative cooling. At 30 s, participants were asked to rate the intensity of coolness, irritation and pleasantness in the mouth. Ratings were made using the oral labelled magnitude scale, adapted from (Cliff and Green 1994a, b; Green et al. 1993). Magnitudes of somaesthetic descriptors were: Barely detectable = 1, weak = 6, moderate = 16, strong = 34, very strong = 50, strongest imaginable = 95 (Green et al. 1993). Data were then presented according to mean intensities. Different formulations were tested at least > 24 h apart to reduce the potential for oral desensitisation.

Study 2 and 3 were comprised of independent participant groups, however, the familiarisation and exercise protocols were the same. Participants were thoroughly briefed on the RPE protocol, as we have previously described (Flood et al. 2017). Before commencing the study, participants conducted a number of RPE-clamp familiarisation tasks to reduced variability. Firstly, participants undertook an RPE ramp test to identify the exercise intensity at each stage associated with the RPE scale. This task was used to calibrate the individual’s understanding of their own RPE. Following a 5‐min rest period, a series of confirmation trials were conducted, starting at 120 W and controlling power output until they achieved an effort that they equated to an RPE-16, across a 3 min period. These trials were continued until the individual could reliably determine the exercise intensity at an RPE-16 within ± 10 W, this took on average 3 attempts. Partial familiarisation of the full experimental RPE-clamp test protocol was then conducted in an environmental heat chamber to experience heat stress and to reduce a subsequent learning effect. Participants were also given significant time to discuss and understand the RPE protocol with the researchers both before and after this initial familiarisation performance trial. Subsequent randomised experimental trials were separated by at least > 72 h and conducted in an environmentally controlled heat chamber, at a temperature of 35.0 ± 0.8 °C and relative humidity 30 ± 3.3%. For each participant, the experimental trials were conducted at the same time of day (± 2 h) to eliminate the effect of circadian variation. All exercise was conducted on an electronically-braked cycle ergometer (Lode Excalibur Sport, The Netherlands).

### Fixed RPE protocol

Upon entering the heat chamber, the participants conducted two standardised RPE warm-up procedures, as used during familiarisation. Participants were then instructed to cycle at a power output that was perceived to represent an RPE of 16 on the 6-to-20 Borg scale (Borg 1982) and to adjust their power output such that an RPE of 16 was maintained. An RPE of 16 represents a verbal cue of between ‘hard’ and ‘very hard’ on the Borg Scale. Participants completed the fixed-RPE protocol and were administered an l-menthol or control mouth rinse immediately prior to and throughout (at 10 min intervals) the experimental protocol. The highest average 30-s power output achieved during the first 3-min of the fixed RPE trial was recorded and participants exercised until their power output declined to 70% of this initial value. The trial was stopped when power output fell below this value for 2-min. Standardised feedback every ~ 2 min was given to remind participants to maintain an RPE of 16. Participants were encouraged to constantly reassess whether they were still exercising at RPE-16. Participants were blinded to distance covered, elapsed time, heart rate, power output.

### Physiological measures

Euhydration was established prior to every trial by identifying urine osmolality < 715 mOsm/Kg H2O (Shirreffs and Maughan 1998) (Pocket Osmochek, Vitech Scientific Ltd, West Sussex, UK) average hydration was 435 ± 188 mOsmols/kg H_2_O. Participants recorded semi-nude body mass (cycling shorts only) prior to entering the heat chamber and immediately following the completion of the experimental trial after wiping off sweat with a towel. No water was ingested during exercise in the heat. Body core temperature was reported using a self-inserted temperature probe (PROACT Medical Ltd. PRTP11112, Northamptonshire, UK) 10 cm past the anal sphincter and logged continuously on a squirrel data logger (Squirrel SQ2040 2F16 data logger, Grant Instruments, Cambridge, UK). Heart rate was recorded continuously throughout the trials (Polar Heart Rate Monitor M400, Warwick, UK).

### Perceptual measures

Participants were thoroughly briefed on the RPE scale before commencing the fixed RPE trials. In addition, participants where familiarised with the thermal sensation (TS) scale which was recorded on an adapted ASHRAE 9-point analogue sensation scale where − 4 = “very cold”, 0 = “neutral”, and 4 = “very hot” (Zhang et al. 2004). Scales were laminated and held in front of the participants to physically indicate scores whilst exercising. Subjective ratings of TS were recorded in 1.0 increments every 10 min during the experimental trials.

### Mouth-rinse formulation

**Fig. 1:**
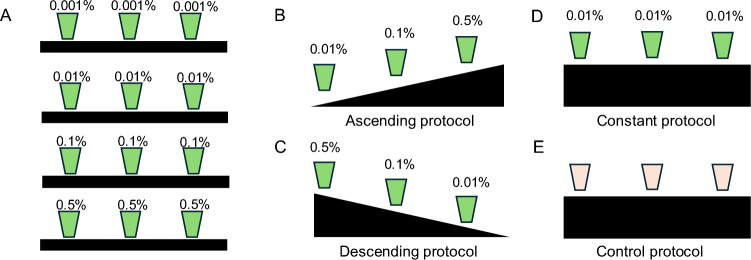
Schematic illustrating the concentration application protocol in study 2 **A** over a range of l-menthol concentrations from 0.001 to 0.5%. In study 3 **B** an ascending protocol used low to high concentrations (0.01–0.5%), **C** a descending protocol used high to low concentrations (0.5–0.01%), **D** a constant protocol applied the standard reported concentration of 0.01% at regular intervals and **E** a control protocol where a strawberry flavoured non-calorific artificial sweetener was applied at regular intervals

Each rinse consisted of a 25 ml colourless solution. The first was given in the 30 s prior to the start of the trial and then at 10-min intervals thereafter. Participants were instructed to swill the solution in their mouth for 10 s before spitting it into a bowl. Five different l-menthol solutions were formulated at a range of concentrations: 0.001%, 0.01%, 0.1%, 0.5% and 1% and compared to a non-calorific flavoured control. In study 2, l-menthol was delivered at each interval at the desired concentration (between 0.001 and 0.5%). In study 3, two differing protocols were used. The descending protocol: 0.5% followed by 0.1% and 0.01% and then 0.01% thereafter until test termination. The ascending protocol: 0.01% followed by 0.1% and 0.5% and then 0.5% thereafter until test termination. The constant protocol: 0.01% delivered throughout (Fig. 1). l-Menthol solutions were made using menthol crystals (≥ 99% food grade l-menthol, Sigma-Aldrich, UK) and first dissolved in mono-propylene glycol (Special Ingredients Ltd, Chesterfield, UK) to facilitate solubilisation and then serial diluted in de-ionized water. To avoid recrystallization of menthol at the highest concentrations, solutions were prepared on a daily basis. A control mouth rinse was formulated using a strawberry flavoured non-calorific artificial sweetener (FlavDrops, MyProtein, Norwich, UK) and mono-propylene glycol, which dissolved in de-ionized water. Solutions were then stored at 5 °C and prior to use warmed back to ~ 31 °C passively in the environmental chamber prior to administration.

### Statistical analysis

All statistical analyses were performed using SPSS (IBM SPSS statistics 22 Inc, USA). A two-way analysis of variance (ANOVA) for repeated measures was used to test for within-group effects across time in all conditions. If sphericity was violated a Greenhouse–Geisser correction was applied. When a significant difference was found for main effect (trial or time), post hoc pair-wise comparisons were made incorporating a Bonferroni adjustment. Magnitude of effect was calculated with partial eta-squared (ηp2) according to the following criteria: 0.02, a small difference; 0. 13, a moderate difference; 0.26 a large difference (Cohen 1988). Differing trial durations meant that power data were normalised with respect to time, and for illustration purposes with respect to starting power output. Perceptual data using the oral labelled magnitude scale was analysed using the non-parametric Friedman test. Data are presented as mean ± SD, significance was set at *P* ≤ 0.05.

## Results

### Study 1—Perception of l-menthol sensory coolness, irritation and pleasantness

**Fig. 2:**
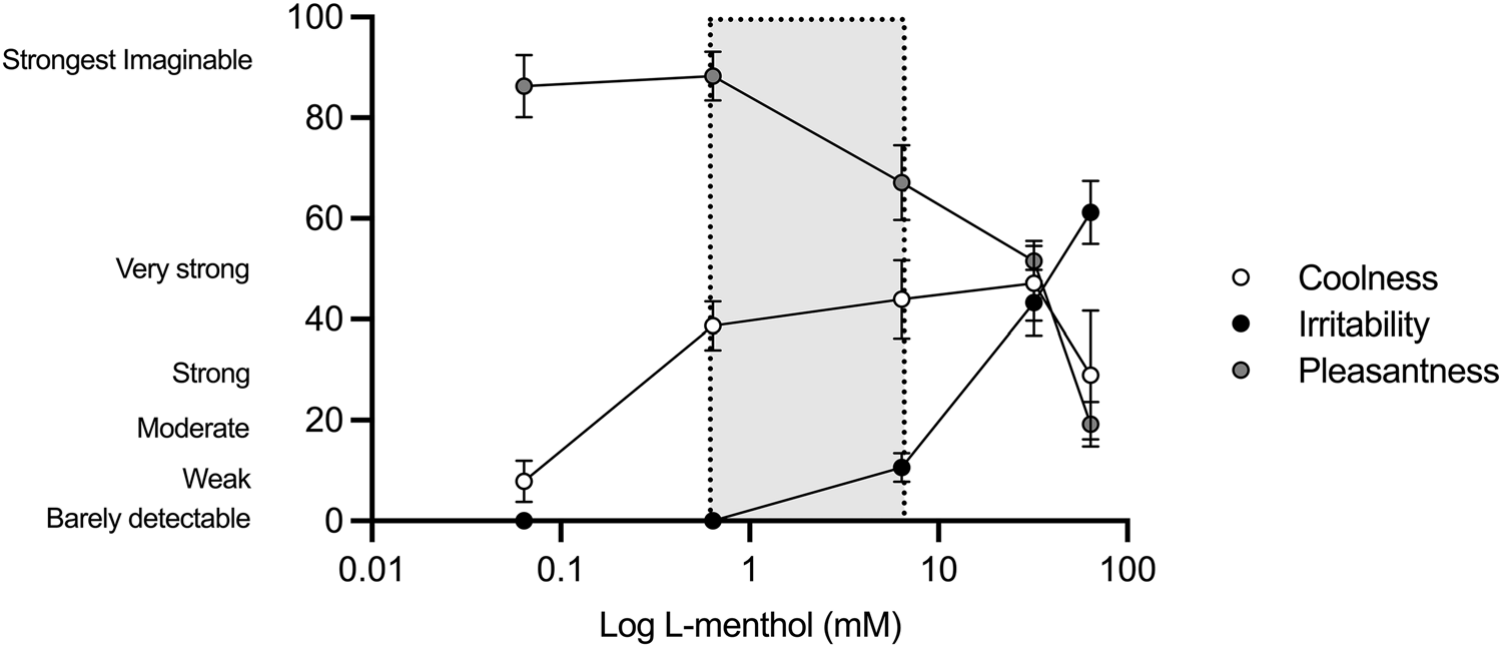
Logarithmic l-menthol concentrations (mM) rated according to the oral labelled magnitude scale with respect to coolness (white circle), irritation (black circle) and pleasantness (grey circle). Data are presented as mean ± SD, *n* = 38. Grey shaded box represents the “optimal” dose range with respect to perceptual and performance data

In study 1, differences were observed across all l-menthol concentrations used for coolness (*z* = 121.59; *P* =  < 0.001), irritation (*z* = 150.40; *P* =  < 0.001) and pleasantness (*z* = 145.99; *P* =  < 0.001). The sensation of coolness was maximised across the dose concentration range of 0.01—0.5% l-menthol, perceived irritation increased at 0.5% l-menthol and reported pleasantness began to decrease beyond a concentration of 0.1% l-menthol (Fig. 2).

### Study 2—Determination of an optimal menthol concentration during exercise

**Fig. 3:**
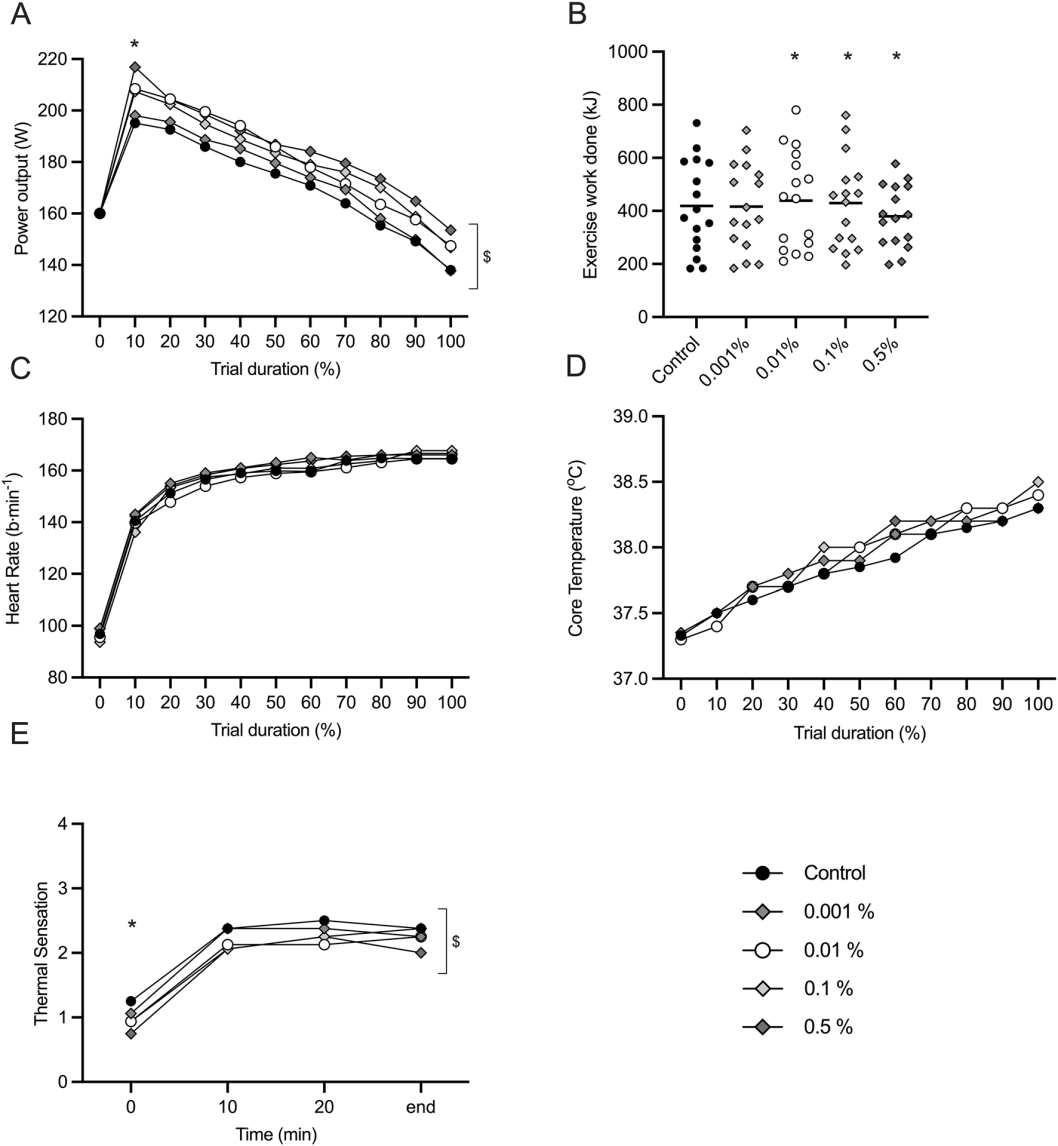
l-Menthol concentration effect on thermo-behaviour exercise modifications, physiology and perception, in a hot environment. **A** Power output; **B** work done; **C** heart rate; **D** body core temperature; **E** thermal sensation. l-menthol concentrations are indicated by colour: control (black), 0.001% (grey, moderate); 0.01% (white), 0.1% (grey, light); 0.5% (grey, dark). Mean data is presented for clarity. * indicates significance differences with respect to control; $ indicates differences between conditions

Power output decreased with time across all conditions (*F*_(2.179, 32.682)_ = 75.788, *P* < 0.001; *η*_*p*_^2^ = 0.835) and was different between conditions (*F*_(4, 60)_ = 3.710, *P* = 0.009; η_p_^2^ = 0.198) (Fig. 2A). The self-determined highest power output achieved was different between conditions (*F*_(4, 60)_ = 2.666, *P* = 0.041; *η*_*p*_^2^ = 0.151), representing ~ 6% increase for 0.01 and 0.1% (*P* < 0.05) and a ~ 9% increase for 0.5% (*P* < 0.05) with respect to control (Fig. 3A). Work completed during trials was different between conditions (*F*_(4, 60)_ = 2.412, *P* = 0.05; *η*_*p*_^2^ = 0.139), relative to control there was no > 1% change at 0.001%, a ~ 5% increase at 0.01% (*P* < 0.05), ~ 3% increase at 0.1% (*P* < 0.05) and a -− 10% decrease at 0.5% (*P* < 0.05) (Fig. 3B). During the exercise task, heart rate increased with time in all conditions (F _(2.021,30.308)_ = 312.242, *P* < 0.001; η_p_^2^ = 0.954) but with no difference between condition (F _(4,60)_ = 1.599, *P* = 0.186; η_p_^2^ = 0.096) (Fig. 3C). At the beginning of all trials, core temperature was not different, averaging 37.3 ± 0.3 °C (*F*_(4, 60)_ = 0.885, *P* = 0.478; *η*_*p*_^2^ = 0.056). During the exercise task, core temperature increased with time in all conditions (*F*_(10, 150)_ = 158.04, *P* < 0.001; *η*_*p*_^2^ = 0.913) but with no difference between condition (*F*_(4, 60)_ = 2.186, *P* = 0.081; *η*_*p*_^2^ = 0.127) (Fig. 3D). Thermal sensation differed across conditions *χ*^2^(19) = 140.893, *P* =  < 0.001 (Fig. 3E). Across the participant sample, there was a ~ 25% (0.3 points) reduction in thermal sensation following administration of the first 0.01% and 0.1% solutions, this was further reduced to ~ 50% (0.6 points) reduction at 0.5%, with respect to control. This was maintained at the 10- and 20-min point for 0.1 and 0.1%, but this difference diminished after 10 min at 0.5%.

### Study 3—Modulating l-menthol concentration during exercise

**Fig. 4:**
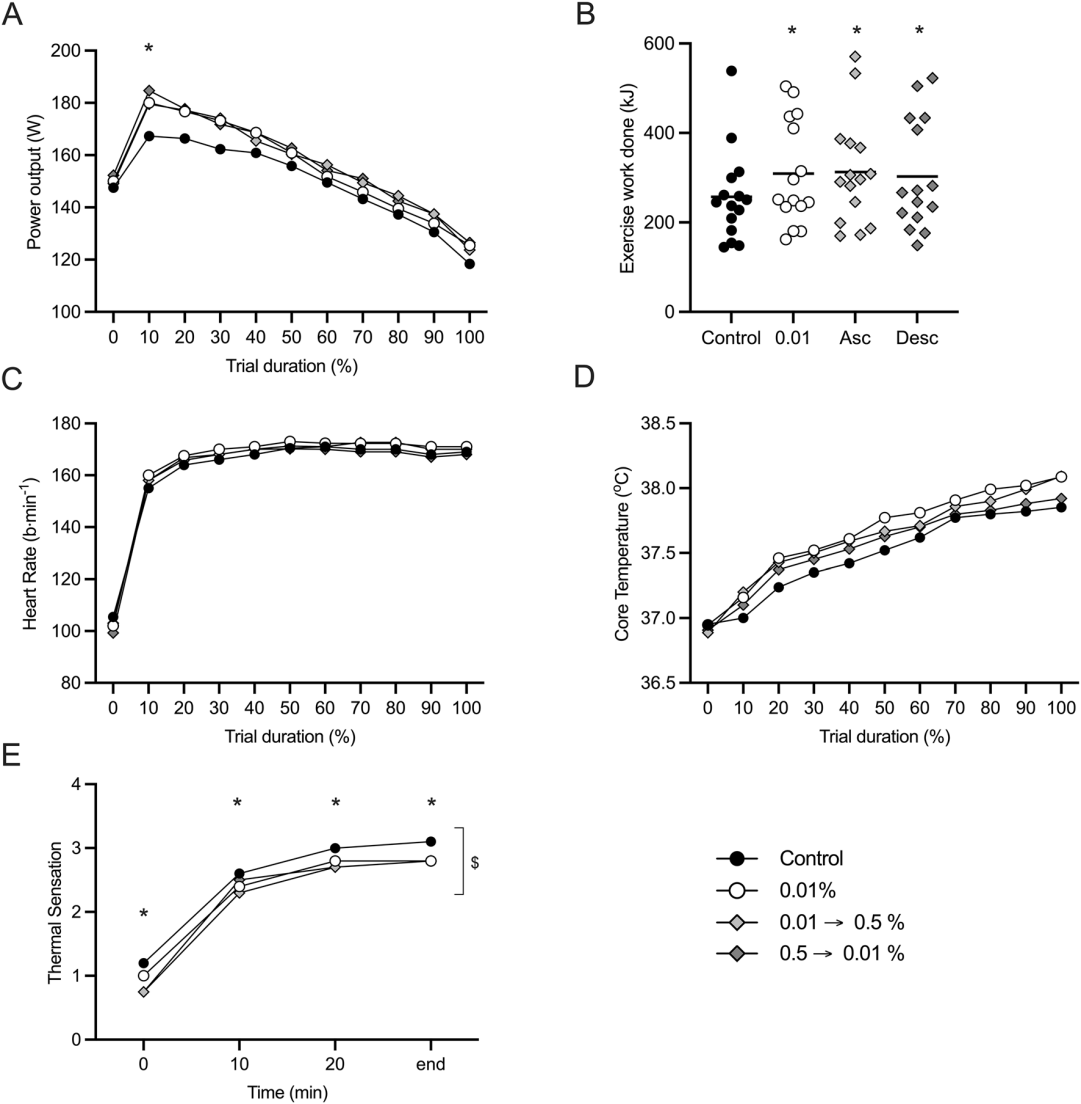
Modulating different l-menthol concentrations during exercise in a hot environment. **A** Power output; **B** work done; **C** heart rate; **D** body core temperature; **E** thermal sensation. l-menthol application strategies are indicated by colour: control (black), constant 0.01% (white), ascending protocol 0.01–0.5% (grey, moderate); descending protocol 0.5–0.01% (grey, dark). Mean data is presented for clarity. * indicates significance differences with respect to control; $ indicates differences between conditions

The self-determined highest power output achieved was different between conditions (*F*_(3, 42)_ = 2.898, *P* = 0.046; *η*_*p*_^2^ = 0.172), representing an equivalent ~ 6% increase in power output across all menthol conditions with respect to control. However, whilst power output decreased with time across all conditions (*F*_(2.565, 35.907)_ = 190.896, *P* < 0.001; *η*_*p*_^2^ = 0.932), there was no difference identified between conditions (*F*_(3, 42)_ = 1.200, *P* = 0.322; *η*_*p*_^2^ = 0.0.79) (Fig. 4A). Work completed during trials was different between conditions (*F*_(3, 42)_ = 2.814, *P* = 0.049; *η*_*p*_^2^ = 0.167), relative to control there was ~ 20% increase in workload for all menthol conditions (Fig. 4B). Heart rate increased with time in all conditions (*F*_(10, 140)_ = 300.106, *P* < 0.001; *η*_*p*_^2^ = 0.955) but with no difference between conditions (*F*_(3, 42)_ = 0.449, *P* = 0.719; *η*_*p*_^2^ = 0.031) (Fig. 4C). At the beginning of all trials, core temperature was not different, averaging 37.0 ± 0.1 °C (*F*_(1.756, 24.585)_ = 0.215, *P* = 0.780; *η*_*p*_^2^ = 0.015). During the exercise task, core temperature increased with time in all conditions (*F*_(1.467, 20.543)_ = 80.781, *P* < 0.001; *η*_*p*_^2^ = 0.852) but with no difference between condition (*F*_(1.781, 24.928)_ = 0.997, *P* = 0.413; *η*_*p*_^2^ = 0.065) (Fig. 4D). Thermal sensation differed across conditions *χ*^2^(15) = 169.010, *P* =  < 0.001 (Fig. 4E). Across the participant sample, there was a ~ 25% (0.3 points) reduction in thermal sensation following administration of the first menthol solution irrespective of concentration, with respect to control, which was largely maintained for the remainder of the trial.

## Discussion

We investigated the effect of a range of l-menthol mouth rinse concentrations on thermo-behavioural exercise responses in a hot environment. We identified an optimal range for application between 0.01 and 0.1%, whilst also identifying that concentrations exceeding 0.5% elicit irritant properties. These effects may impair l-menthol’s properties as a non-physical perceptual coolant, by instead acting as an irritant. Further, altering the l-menthol concentration from lower to higher did not enhance subsequent applications of l-menthol on exercise work rate. Therefore, individuals may only have one attempt to avail of l-menthol’s ergogenic cooling properties in the heat applied at a concentration between 0.01% and 0.1%.

### Determination of an optimal l-menthol concentration

The RPE-clamp exercise protocol enables self-determination of work rate to be observed according to a level of perceived exertion (in this case RPE = 16, described as between hard and very hard). The rate of heat storage during exercise is correlated with an anticipatory reduction in RPE controlled work rate (Tucker et al. 2006) and, therefore, is a useful model to observe the impact of changes in thermal perception to manage heat stress. We replicated our previous findings using the same protocol (Flood et al. 2017), where mouth rinsing with a 0.01% l-menthol solution elicited a ~ 5% increase in total work completed, with respect to control. However, an optimal concentration of l-menthol has not been previously established. Here, we found that lower concentrations of 0.001% l-menthol elicited no change > 1% in exercise work. At higher concentrations of 0.1% l-menthol, a smaller change of ~ 2.5% above control was observed. At the highest concentration utilised, 0.5% l-menthol, total work completed was in fact reduced by -10%. However, these observations on total work completed contrast the initial peak power freely selected by participants at the beginning of the trial. Peak power achieved was comparable at 0.01% and 0.1% l-menthol, equating to a ~ 6% increase with respect to control. At a lower concentration of 0.001% l-menthol, there were no differences with respect to control (> 1%). Intriguingly, at a higher 0.5% l-menthol concentration, a ~ 10% increase in peak power was observed. Hence, at the highest concentration of 0.5% l-menthol there was a marginal increase in peak power but, as a result, there was an overall decrease in performance across the test with respect to 0.01% and 0.1% l-menthol solutions. It is plausible that higher concentrations may impact the anticipatory determination of workload, facilitating the adoption of a higher initial power output associated with RPE = 16, but which ultimately, across an endurance trial, facilitates a faster onset of fatigue. Whilst we have already established l-menthol’s effectiveness at extending exercise performance in the heat and have suggested this may be mediated by corresponding reductions in thermal sensation (Jeffries and Waldron 2019), we have also previously explored alternate explanations (Jeffries et al. 2018). Individuals reported greater irritation and unpleasantness at higher 0.5% l-menthol concentrations, which suggests that some of the cooling properties may be diminished. It is possible that l-menthol’s ergogenic power, either across the concentration range presented or selectively at higher concentrations, may be in its role as a distractor or irritant, rather than a cooling stimulus per se. Indeed, we have previously observed a greater frequency of distractions following topical application of an l-menthol cream during an exhaustive exercise task in the heat (Peel et al. 2023). Distraction could facilitate a reallocation of attention away from stressful thermal and physiological stimuli (Brick et al. 2014) towards taste sensation, which may be advantageous during endurance exercise, but could be specifically advantageous during brief episodes thus enabling a higher self-determination of power output associated with a fixed RPE. Such redirection of attention can offset fatiguability thereby enhancing performance (Lohse and Sherwood 2011), and strong taste sensations may elicit dissociative thoughts capable of distracting attention away from the sensations associated with exercise (Baden et al. 2004). Indeed, unpleasant bitter solutions such as quinine have been shown to positively alter exercise performance, albeit through a different molecular target (Gam et al. 2016). However, it is clear that such disinhibition which facilitated the achievement of a greater peak power, negatively affected work completed across the remainder of the trial.

Consistent with the menthol literature, there were no discernible differences in cardiovascular measures of heart rate or body core temperature between conditions. However, we did not measure ventilatory parameters or permit fluid intake during the trials which could have facilitated further discrimination between the l-menthol concentrations used. l-menthol’s ability to enhance cooling of the upper airways may have influenced breathing parameters or influenced fluid intake modulating thermo-behaviour during the RPE-clamp protocol. Thermal sensation was clearly reduced in 0.01 and 0.1% and further reduced in 0.5% consistent with l-menthol’s role in modulating thermal perception in hot environments, although these differences largely diminished ~ 20 min into the trials. We can therefore conclude that a concentration range between 0.01 and 0.1% l-menthol are optimal to elicit positive changes in perceived thermal sensation and increase work completed in hot environments.

### In search for the optimal dose

**Fig. 5:**
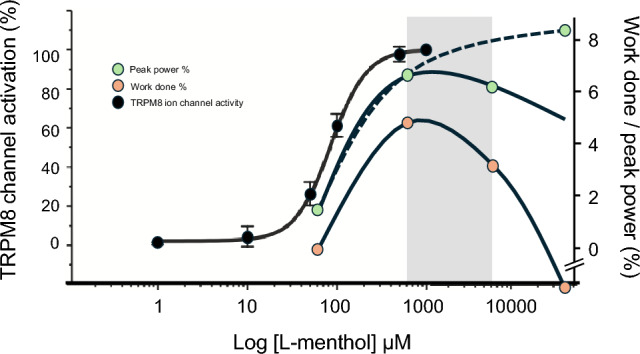
Determination of l-menthol’s optimal dose. l-menthol’s molecular target TRPM8 channel activation data (black circles) as a % of total activation (data presented is adapted from McKemy et al. 2002). l-menthol ergogenic modulation of exercise peak power associated with RPE-16 (green circles) and total work at RPE-16 (red circles), as a % above baseline placebo tests. Grey shaded box represents the “optimal” performance range. Dashed line represents l-menthol’s alternative actions as an irritant, possibly evoking increase in performance. Exercise was completed in 35 °C/40 RH% environment

In cellular models exploring TRPM8 channel activation, the half maximal effective concentration (EC_50_) for l-menthol is reported in the nanomolar to low micromolar range. McKemy et al. (2002), who first cloned the TRPM8 temperature sensitive channel in 2002, reported that menthol activated the isolated channel with an EC_50_ ~ 80 µM, equivalent to a concentration dose of ~ 0.0013% (McKemy et al. 2002). Indeed, a plateau in channel activity can be observed < 1 mM, equivalent to a concentration dose ~ 0.016% (McKemy et al. 2002) (Fig. 5). The range of menthol concentrations typically reported in the literature have molar concentrations ranging between 0.6 and 6 mM, equivalent to a 0.01 and 0.1% solution, respectively. These applied concentrations are on the upper portion of the dose response curve and if one were to assume full oral receptor occupancy during a 10 s mouth rinse, then very much at its maximal effective dose (Fig. 5). The lower concentration examined in this study was equivalent to a 0.001% solution, translating to a molar concentration of 64 µM, which is below the EC_50_ for channel activation. This perhaps explains why this concentration was less effective in eliciting a thermo-behavioural response and matched the control solutions that were administered (Fig. 5). Optimal reported coolness without irritation, alongside optimal exercise work performed, occurs in the range 0.01–0.1% (see Figs. 3, 5 grey shaded box), with higher concentrations reducing workload and increasing irritation and unpleasantness. In contrast, higher concentrations may have been beneficial in eliciting peak power, possibly via an alternate mechanism. This may be attributed to l-menthol’s multi-modal properties to elicit thermal cooling via TRPM8 (McKemy et al. 2002) and irritation/burning via TRPA1/TRPV1 (Julius 2013), the latter eliciting a different stimulus to that traditionally associated with l-menthol (see Fig. 5, green circle dashed line).

### Changing strategy, modulating l-menthol concentration during exercise

We previously speculated that l-menthol’s ability to modulate thermal sensation, altering exercise strategy, might dissipate as a function of exercising heat exposure (Flood et al. 2017). We furthered this work establishing that in the latter stages of exercise in a hot environment, during advanced internal thermal strain (~ 38.5 °C), l-menthol was still effective at extending performance by ~ 6% (Jeffries et al. 2018). However, it remained unclear whether l-menthol could be utilised on more than one occasion and whether modulating the applied concentration could facilitate enhanced ergogenic potential. Here, we can confirm that modulating dose from the lowest effective dose (0.01%) to the highest tolerable dose (0.5%), and reversing this dose strategy, had no effect with regard to the downward trajectory of work rate associated with an RPE-16. Indeed, neither strategy was more or less ergogenic than applying 0.01% consistently throughout the trial. However, all menthol application strategies were equally ergogenic in increasing work done by ~ 20% with respect to control, in this participant group. Further, no differences in cardiovascular measures of heart rate or body core temperature were observed between conditions. Thermal sensation was also reduced ~ 25% following administration of any menthol solution with respect to control, which was largely maintained, in this study, for the duration of the trial. We can, therefore, conclude that varying l-menthol concentration is unable to augment the effectiveness of a first application and maintain performance in an RPE-clamp exercise task. We are unable to clearly state whether individuals are desensitised to subsequent applications of l-menthol irrespective of concentrations. However, in vitro studies in isolated cells have demonstrated that TRPM8 channels, the key molecular target for l-menthol in the oral cavity, will be desensitised to l-menthol application by as much as ~ 55% upon secondary activation (McKemy et al. 2002). This may explain l-menthol’s diminishing ergogenic properties particularly in the event of increasing heat stress and exercise fatigue. Whilst we have applied l-menthol at 10-min intervals throughout the exercise task, further studies have reported in rat models that the oral cavity remains insensitive to l-menthol for up to 10-min after application (Lundy and Contreras 1993). However, it remains unknown whether a longer period between l-menthol applications would allow individuals to utilise l-menthol on more than one occasion.

## Conclusion

From the evidence gathered, to date, from our laboratory and in the studies presented, the optimal dose of l-menthol when delivered via oral rinsing is within the range of 0.01–0.1%. At lower concentrations, l-menthol appears to be less effective and not different from control solutions, and at higher concentrations (> 0.5%), l-menthol appears to elicit greater irritation and may not positively modulate thermo-behaviour during exercise in a hot environment. However, while the current data indicate that the chosen method of incremental or decremental application does not enhance the effect of a single ergogenic concentration, it remains unclear whether repeated applications can elicit subsequent ergogenic effects with different timings.

## Data Availability

The datasets generated during and/or analyzed during the current study are available from the corresponding author upon reasonable request.
